# Case Report: A successful rechallenge with aumolertinib after osimertinib-induced severe interstitial lung disease

**DOI:** 10.3389/fphar.2026.1785616

**Published:** 2026-04-13

**Authors:** Hejing Bao, Caijiu Deng, Xi Luo, Haoran Su, Hehong Bao, Jiazhu Hu, Xiaolong Cao, Liping Lin

**Affiliations:** 1 Department of Oncology, The Affiliated Panyu Center Hospital, Guangzhou Medical University, Guangzhou, Guangdong, China; 2 Cancer Institute of Panyu District, The Affiliated Panyu Center Hospital, Guangzhou Medical University, Guangzhou, Guangdong, China; 3 Department of Psychosomatic Medicine, Chongqing University Three Gorges Hospital, Chongqing, China

**Keywords:** aumolertinib, EGFR-TKI, interstitial lung disease, non-small cell lung cancer, osimertinib

## Abstract

**Background:**

Osimertinib, a third-generation epidermal growth factor receptor (EGFR) tyrosine kinase inhibitor (TKI), demonstrates significant efficacy in treating non-small cell lung cancer (NSCLC) harboring sensitive EGFR mutations. However, EGFR-TKI-induced interstitial lung disease (ILD) is a recognized and severe adverse reaction that can be fatal. Given the often limited patient acceptance of chemotherapy, there is currently no international consensus on the efficacy and safety of rechallenging with an EGFR-TKI following EGFR-TKI-induced ILD.

**Case Summary:**

We report the case of a 73-year-old male with stage IV lung adenocarcinoma carrying an EGFR exon 19 deletions (Ex19del) mutation, who received first-line osimertinib at 80 mg daily. In the third month of treatment, the patient developed grade IV (CTCAE v5.0) ILD, presenting with dyspnea, chest tightness, dry cough, and fever. Arterial blood gas analysis indicated type I respiratory failure, and a chest CT scan revealed new bilateral patchy and reticular opacities. As high-flow nasal cannula oxygen failed to maintain adequate oxygen saturation, the patient was transferred to the intensive care unit (ICU), where he received endotracheal intubation, anti-inflammatory therapy with methylprednisolone, and anti-infective treatment. After 18 days, he recovered well and was discharged. Post-discharge, oral corticosteroid therapy was continued, and nintedanib was administered for its anti-fibrotic effects. The patient subsequently declined chemotherapy. Two months after corticosteroid therapy, EGFR-TKI treatment was rechallenged with aumolertinib at a reduced dose of 55 mg daily. After 1 month of treatment, the patient experienced no recurrence of dyspnea or other respiratory symptoms. A follow-up CT scan indicated resolution of the interstitial pneumonia and shrinkage of the pulmonary tumor. The aumolertinib dose was then increased to the standard 110 mg daily and treatment was continued.

**Conclusion:**

Rechallenging with an EGFR-TKI after osimertinib-induced ILD remains highly challenging, particularly for grade 3 or higher ILD. In this case, following an adequate course of corticosteroid and anti-fibrotic therapy, successful rechallenge was achieved by initiating treatment with a low dose of aumolertinib, which was later escalated to the conventional dose.

## Introduction

Lung cancer remains the leading cause of cancer-related mortality, with NSCLC representing the most prevalent histological type (Siegel et al., 2025; Lee et al., 2024). NSCLC, encompassing adenocarcinoma and squamous cell carcinoma, accounts for 80%–90% of all lung cancer diagnoses. Approximately 60% of patients present with advanced or metastatic disease at diagnosis (Siegel et al., 2025; Lee et al., 2024). In these cases, activating mutations in the EGFR gene are found in approximately 10% of lung adenocarcinomas in European/North American populations, and in up to 50%–60% of cases in East Asian populations (Al Bakir et al., 2025; Ciardiello et al., 2024). EGFR TKIs are the preferred first-line treatment for NSCLC harboring sensitizing EGFR mutations, primarily Ex19del and the L858R point mutation (Fu et al., 2022; Grant and Nagasaka, 2024).

The development and clinical application of EGFR-TKIs have significantly improved survival and clinical outcomes for patients with EGFR-mutant NSCLC. Consequently, these agents are endorsed by major treatment guidelines, including those from the National Comprehensive Cancer Network (NCCN), as standard first-line therapy (Fukuoka et al., 2011; Maemondo et al., 2010; Soria et al., 2018). Approved first-generation EGFR-TKIs, such as gefitinib and erlotinib, act by reversibly binding to the ATP-binding pocket of the EGFR tyrosine kinase domain, effectively inhibiting receptor activation and cellular proliferation (Sabbah et al., 2020; Shi et al., 2025). Second-generation agents, including afatinib, covalently and irreversibly bind to EGFR, demonstrating superior efficacy in some settings compared to first-generation TKIs (Sabbah et al., 2020; Shi et al., 2025; Sabbah et al., 2020). Third-generation EGFR-TKIs, exemplified by osimertinib, were developed to overcome the acquired T790M resistance mutation. They form a stable covalent bond with EGFR, including the T790M mutant variant, offering a targeted therapeutic strategy for resistant disease (Akamatsu et al., 2021; Planchard et al., 2023).

Despite their efficacy, all generations of EGFR-TKIs are associated with toxicities. The most common adverse events with osimertinib include rash, diarrhea, and dry skin (Soria et al., 2018; Herbst et al., 2023). However, a serious and potentially fatal class effect is drug-induced ILD. According to the NCI-CTCAE version 5.0 criteria, ILD severity is graded from 1 to 5, with grade 3 or higher considered severe. Grade IV ILD, requiring mechanical ventilation, carries a mortality rate as high as 30%–50% (Yin et al., 2022). Diagnosis relies on a combination of clinical symptoms (e.g., progressive dyspnea, dry cough), characteristic imaging findings, and bronchoscopy to exclude infectious etiologies via bronchoalveolar lavage fluid (BALF) analysis. The incidence of EGFR-TKI-related ILD varies among agents, ranging from 0% to 5.3% (Yin et al., 2022). For osimertinib, the reported incidence is approximately 3%–4%, but it can be as high as 8%–12% in Japanese populations. Notably, there have been reports of failed rechallenge attempts following osimertinib-induced grade IV ILD.

Aumolertinib (formerly known as aumolertinib) is a domestically developed third-generation EGFR-TKI in China. It exhibits high selectivity for EGFR sensitizing mutations and the T790M resistance mutation. Its pharmacokinetic profile, characterized by a long half-life (30–50 h), supports once-daily dosing. In pivotal clinical trials (APOLLO, AENEAS), the incidence of aumolertinib-related ILD was approximately 1.5%–1.9%, with no reported cases of grade ≥3 ILD, suggesting a potentially more favorable pulmonary safety profile (Lu et al., 2022a; Lu et al., 2022b). However, data on the use of aumolertinib following recovery from osimertinib-induced severe (grade IV) ILD are extremely scarce. Herein, we report a successful rechallenge case with aumolertinib in a patient with EGFR Ex19del lung adenocarcinoma who recovered from osimertinib-induced grade IV ILD.

## Case presentation

**FIGURE 1:**
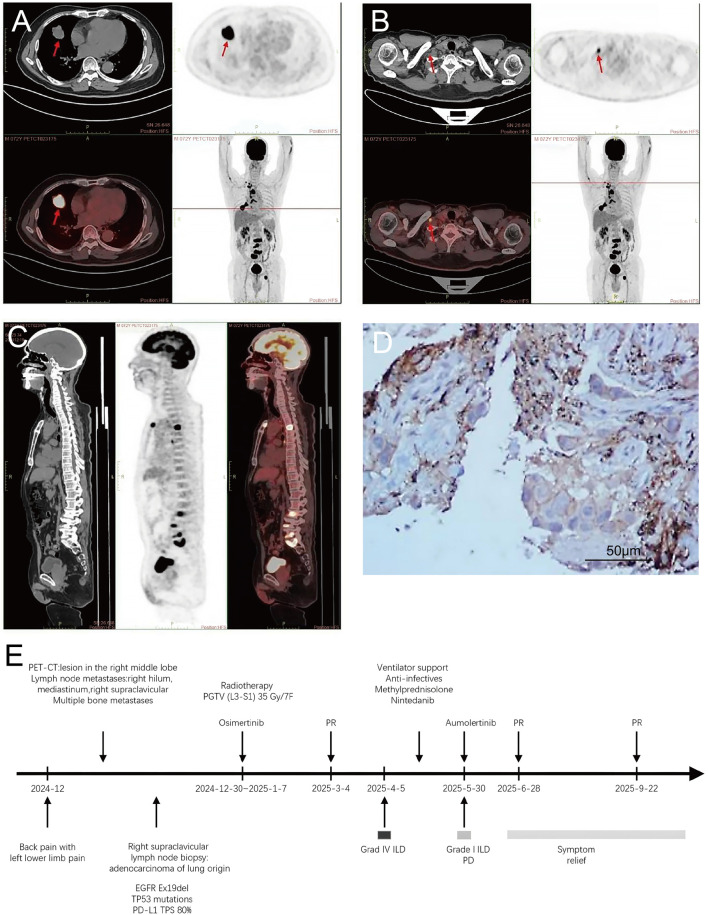
Patient’s Clinical Course and Baseline Characteristics. **(A)** PET-CT image showing an irregular soft-tissue mass with increased metabolism in the lateral segment of the right middle lobe (approximately 30 × 39 × 28 mm), suggestive of peripheral lung cancer with peritumoral inflammation. **(B)** PET-CT image demonstrating a hypermetabolic metastatic lymph node in the right supraclavicular region. **(C)** PET-CT image revealing multiple osteolytic bone lesions throughout the skeleton, indicative of bone metastases, with lumbar spine involvement and partial pathological fracture. **(D)** Immunohistochemical staining for PD-L1 expression (Dako 22C3 antibody) of the right supraclavicular lymph node biopsy, showing a TPS of 80% (original magnification, ×400). **(E)** Flowchart summarizing the patient’s treatment timeline.

A 73-year-old Chinese male, residing in an urban area of southern China, presented with a significant smoking history of 30 pack-years (current smoker). He denied any history of occupational dust exposure. His medical history included hypertension, which was well-controlled with medication. Notably, his father had a history of lung cancer. In December 2024, the patient presented to our hospital with lower back pain and left lower limb pain. A lumbar spine CT performed on 17 December 2024, revealed multiple osteolytic bone lesions at L3-S1, suggestive of metastases, accompanied by a pathological fracture of the L3 vertebra. A thoracolumbar MRI on 18 December 2024, confirmed abnormal signals in the T4, L3-5, and S1 vertebral bodies, consistent with bone metastases and the L3 pathological fracture. A subsequent PET-CT scan on 20 December 2024, demonstrated: 1) An irregular soft-tissue mass (approximately 30 × 39 × 28 mm) with increased metabolism in the lateral segment of the right middle lobe, suggestive of peripheral lung cancer with peritumoral inflammation (Figure 1A). 2) Multiple hypermetabolic lymph nodes in the right hilum, mediastinum (stations 1R, 2, 3, 4R, 7), right supraclavicular region, and subcutaneous tissue of the right upper arm, indicative of metastases (Figure 1B). 3) Multiple small pulmonary nodules in both lungs without significant metabolism, some suspected of being metastases. 4) Swelling of the right quadratus lumborum muscle with small nodular/patchy hypermetabolic foci, suspicious for metastasis. 5) Widespread osteolytic bone lesions with increased metabolism throughout the skeleton, consistent with bone metastases and partial pathological fractures (Figure 1C).

**FIGURE 2:**
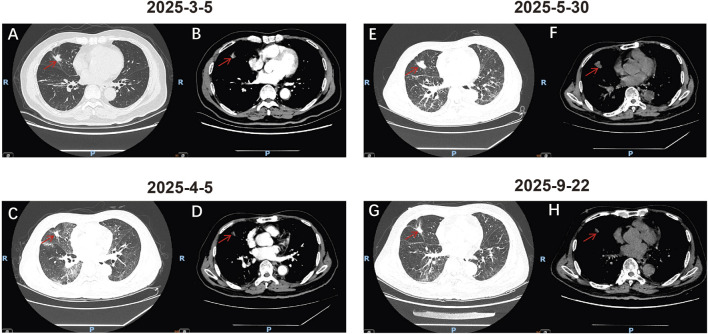
Radiological Assessment of the Primary Tumor. **(A,B)** Chest CT images on 5 March 2025, showing the primary lesion in the lateral segment of the right middle lobe, measuring approximately 17 × 13 mm after treatment for bone metastases. **(A)** Lung window. **(B)** Mediastinal window. **(C,D)** Chest CT images on 5 April 2025, showing a slight reduction in the size of the primary lesion to approximately 15 × 10 mm. **(C)** Lung window. **(D)** Mediastinal window. **(E,F)** Chest CT images on 30 May 2025, demonstrating an increase in the size of the primary lesion to approximately 24 × 16 mm. **(E)** Lung window. **(F)** Mediastinal window. **(G,H)** Chest CT images on 22 September 2025, showing a reduction in the size of the primary lesion to approximately 16 × 13 mm following aumolertinib rechallenge. **(G)** Lung window. **(H)** Mediastinal window.

**FIGURE 3:**
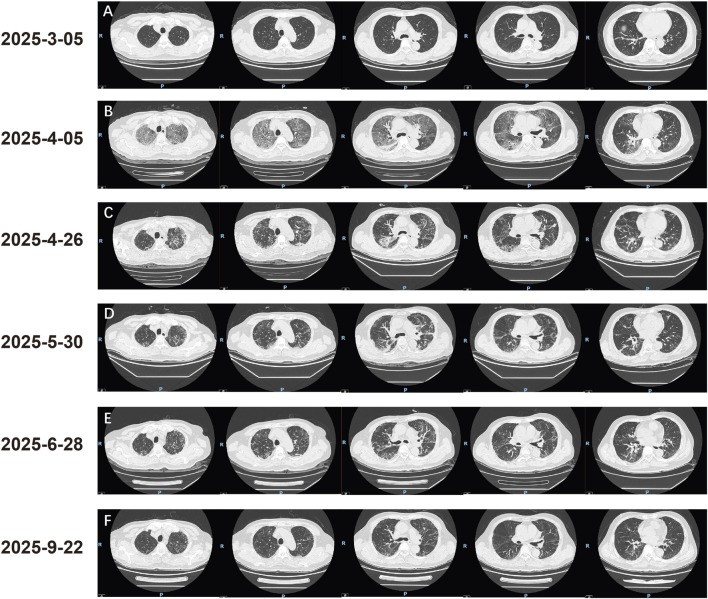
Evolution of ILD. **(A)** Chest CT on 5 March 2025, 2 months after initiating osimertinib, showing no evidence of ILD. **(B)** Chest CT on 5 April 2025, 3 months after osimertinib initiation, revealing new bilateral interstitial pneumonia, predominantly in the upper lobes, involving more than 50% of the lung fields. **(C)** Chest CT on 26 April 2025, 3 weeks after ILD treatment, showing scattered lung infiltrates with interstitial thickening; some lesions in the lower lobes had progressed compared to prior imaging. **(D)** Chest CT on 30 May 2025, approximately 8 weeks after ILD treatment, demonstrating significant resolution of pneumonia. The primary tumor showed enlargement compared to previous scans. **(E)** Chest CT on 28 June 2025, approximately 4 weeks after initiating aumolertinib rechallenge, showing continued resolution of inflammation and reduction in tumor size. **(F)** Chest CT on 22 September 2025, approximately 3 months after aumolertinib rechallenge, showing no signs of recurrent pneumonia and further tumor shrinkage.

A biopsy of a right supraclavicular lymph node was performed on 23 December 2024. Pathology confirmed metastatic, moderately to poorly differentiated lung adenocarcinoma. Immunohistochemistry was positive for CK7 and TTF-1, and negative for p40 and NapsinA. Genetic testing revealed an EGFR Ex19del (20.55%), a TP53 exon 7 missense mutation (5.74%), and a PD-L1 Tumor Proportion Score (TPS) of 80% (Figure 1D). The serum carcinoembryonic antigen (CEA) level was elevated at 231.00 ng/mL. According to the eighth edition of the American Joint Committee on Cancer (AJCC) staging system, the patient was diagnosed with stage IV (T2N3M1) right lung adenocarcinoma. The patient subsequently received palliative radiotherapy (35 Gy in 7 fractions) to the L3-S1 vertebral metastases from 30 December 2024, to 7 January 2025. First-line targeted therapy with osimertinib 80 mg once daily was initiated on 2 January 2025. Subsequent imaging evaluations indicated a partial response (PR) to treatment (Figures 2A,B, 3A).

However, on 5 April 2025, the patient was admitted with new-onset dry cough, chest tightness, exertional dyspnea, generalized myalgia, fatigue, and anorexia. Laboratory tests showed an elevated C-reactive protein (86.99 mg/L) and procalcitonin (0.12 ng/mL). Despite initial empiric antibiotic therapy, his respiratory status deteriorated, with a peripheral oxygen saturation of only 90% despite receiving 9 L/min of oxygen via nasal cannula. A chest CT revealed new bilateral, patchy, and reticular opacities with ground-glass attenuation and interlobular septal thickening, predominantly in the upper lobes, consistent with ILD (Figure 3B). The primary lung lesion appeared slightly smaller (Figures 2C,D). Arterial blood gas analysis revealed type I respiratory failure with a PaO_2_/FiO_2_ ratio of 119.3. Due to progressive hypoxemia, the patient was intubated and transferred to the ICU for mechanical ventilation. In the ICU, bronchoalveolar lavage (BAL) was performed. Pathogen detection via tNGS identified *Acinetobacter* baumanniiand HSV-1, both with low estimated concentrations (<1.0E+3 copies/mL). Serological tests for autoimmune etiologies (cANCA, pANCA, PR3/MPO antibodies, antinuclear antibody) were negative. There was no clinical evidence of heart failure, and the patient was not taking other medications known to induce ILD. Based on the clinical presentation, characteristic imaging findings, and exclusion of other causes, osimertinib was determined to be the cause of grade 4 (CTCAE v5.0) ILD.

Osimertinib was immediately discontinued. The patient was treated with methylprednisolone 80 mg daily and continued on prophylactic antibiotics. His oxygenation improved gradually with corticosteroid therapy. He was successfully extubated on the fourth day after intubation and transferred back to the general oncology ward. The corticosteroid dose was gradually tapered over 2 months following discharge. Concurrently, the patient received nintedanib 150 mg twice daily for its anti-fibrotic effects. A follow-up CT on 30 May 2025, showed significant resolution of the interstitial pneumonia (Figure 3D). However, concurrent imaging revealed enlargement of the primary tumor (Figures 2E,F) and a rising CEA level, indicating tumor progression. We engaged in detailed shared decision-making with the patient and his family, discussing alternative options including platinum-based chemotherapy with pemetrexed, single-agent docetaxel, best supportive care, and clinical trial enrollment. The patient, concerned about chemotherapy-related toxicities (e.g., alopecia, gastrointestinal effects) and his fragile condition post-severe ILD, explicitly declined chemotherapy. He expressed a strong preference to continue targeted therapy, fully understanding the risk of ILD recurrence with rechallenge. After thorough discussion of the potential risks and benefits, the patient provided written informed consent specifically for the rechallenge attempt.

Consequently, on 30 May 2025 (93 days after ILD onset), rechallenge with a reduced dose of aumolertinib 55 mg once daily was initiated. Follow-up CT scans on 28 June 2025 (Figure 3E), and 22 September 2025, demonstrated tumor regression (Figures 2G,H, 3F) without recurrence or worsening of ILD. The aumolertinib dose was subsequently increased to the standard 110 mg once daily, while nintedanib was continued. Over the subsequent 7 months of aumolertinib treatment, the patient experienced no ILD recurrence. Treatment-related adverse events were limited to grade 1 rash and diarrhea, which were managed with supportive care. The patient’s treatment timeline is summarized in a flowchart (Figure 1E).

## Discussion

This study reports a successful rechallenge with aumolertinib in a patient with advanced lung adenocarcinoma harboring an EGFR Ex19del mutation, who had recovered from osimertinib-induced life-threatening grade 4 ILD. Successful rechallenge was achieved through a comprehensive management strategy involving a full course of corticosteroid therapy combined with the anti-fibrotic agent nintedanib, followed by the initiation of aumolertinib at a reduced dose with subsequent escalation. This approach resulted in favorable tumor control. Our case provides valuable clinical insights into the management strategy following severe EGFR-TKI-induced ILD.

EGFR-TKIs are the standard first-line therapy for advanced EGFR-mutant NSCLC. However, EGFR-TKI-induced ILD, though uncommon, is a potentially fatal adverse event (Soria et al., 2018; Yin et al., 2022). While the incidence of ILD is relatively lower with third-generation agents like osimertinib, its occurrence, particularly at high grades (≥ grade 3), is often severe, associated with high mortality, and drastically limits subsequent treatment options (Soria et al., 2018). Currently, there is no international consensus on whether and how to rechallenge with an EGFR-TKI after a related ILD event. The prevailing view advocates extreme caution regarding rechallenge after severe ILD, often recommending permanent discontinuation of the offending TKI and a switch to alternative therapies such as chemotherapy, due to the high risk of life-threatening ILD recurrence (Tachi et al., 2017; Gu et al., 2024; Jiang et al., 2021). Although successful rechallenge cases, with or without concomitant corticosteroids, have been reported (Kodama et al., 2021; Miyauchi et al., 2017; Nishima et al., 2021; Nagasaka and Gadgeel, 2018; Kiriu et al., 2018; Kanaji et al., 2024), the patient in the present case had low acceptance of chemotherapy, necessitating the exploration of a more challenging therapeutic pathway.

The success in this case may be attributed to several key factors. First, adequate management of the acute ILD phase and facilitation of lung repair formed the essential foundation for rechallenge. Corticosteroids, due to their anti-inflammatory and immunosuppressive properties, are commonly employed as first-line therapy for ILD (Van den Bosch et al., 2022). They inhibit leukocyte migration and infiltration into inflamed tissues, interfere with the functions of leukocytes, fibroblasts, and endothelial cells, and suppress humoral mediators (Johnson et al., 2023). In fibrotic ILDs, while complete reversal is not possible, short-term corticosteroids play a role in stabilizing rapidly progressive disease (Johnson et al., 2023). For grade 3 or higher ILD, an initial dose of prednisolone 1.0–2.0 mg/kg/day (or equivalent) for 2–4 weeks is recommended until clinical and radiographic improvement, followed by a slow taper over a total course of at least 8 weeks (Chinese Society of Lung Cancer, 2019). For grade 4 ILD, pulse therapy with methylprednisolone 500–1000 mg/day for 3 days is even suggested (Chinese Society of Lung Cancer, 2019). In our patient, upon diagnosis of grade 4 ILD, osimertinib was immediately and permanently discontinued. The patient received a sufficient dose and full course of corticosteroid therapy, effectively suppressing the immune-inflammatory response. Combined with broad-spectrum anti-infective therapy, this allowed successful weaning from mechanical ventilation on the fourth day. The subsequent corticosteroid taper was conducted cautiously over a prolonged period to ensure efficacy while mitigating common adverse effects.

More importantly, we concurrently administered the anti-fibrotic agent nintedanib during corticosteroid therapy. Nintedanib is a multiple tyrosine kinase inhibitor that targets pathways including platelet-derived growth factor receptor (PDGFR), fibroblast growth factor receptor (FGFR), and vascular endothelial growth factor receptor (VEGFR), thereby inhibiting fibroblast proliferation, migration, and transformation, and slowing pulmonary fibrosis progression (Maher, 2024). Nintedanib is approved for the treatment of systemic sclerosis-associated ILD (SSc-ILD) and, based on the SENSCIS and INBUILD trials, for chronic fibrosing ILDs with a progressive phenotype (Flaherty et al., 2019; Distler et al., 2019). Following the acute inflammatory phase of ILD, aberrant repair of the alveolar epithelium often leads to fibrosis (Ohmori et al., 2021; Wong et al., 2020), which may be a risk factor for persistent lung function impairment and rechallenge failure. The early introduction of nintedanib in our case may have effectively mitigated fibrotic repair in the lung tissue, providing a better structural basis for functional recovery and subsequent tolerance to TKI therapy.

The precise mechanisms underlying EGFR-TKI-induced ILD remain incompletely understood but are thought to involve lung injury, fibrosis, and drug-triggered immune responses. EGFR-TKIs block EGFR phosphorylation, thereby preventing the regeneration of damaged epithelium and impeding the repair process (Ohmori et al., 2021). This disruption of the injury-repair mechanism regulated by EGFR activation may contribute to fatal lung injury (Wong et al., 2020). Furthermore, EGFR-TKIs may induce pulmonary fibrosis by stimulating fibroblast migration and proliferation (Matsuno, 2012). They can disrupt the balance of cell survival under TNFα-rich conditions via inhibition of protein kinase B (AKT) and extracellular signal-regulated kinase (ERK)1/2, and activation of p38 MAP kinase, leading to pulmonary inflammation and induction of apoptosis (Yamaoka et al., 2019). Additionally, studies suggest that EGFR-TKIs may inhibit heat shock protein 70 (HSP-70) expression in the lung, potentially promoting pulmonary fibrosis (Enomoto, 2022).

In this case, we opted to rechallenge with aumolertinib rather than resuming osimertinib or switching to a TKI from a different class. This decision was based on multiple considerations, including the existing data on the incidence of ILD among third-generation EGFR-TKIs. Real-world pharmacovigilance analyses have reported varying ILD rates. In the FAERS database, osimertinib was associated with the highest absolute number of reported ILD cases (156 cases) (Miao et al., 2025). In contrast, aumolertinib has demonstrated a relatively favorable pulmonary safety profile in Chinese clinical trials and post-marketing surveillance, with an estimated ILD incidence of 1%–2% (Lu et al., 2022a; Lu et al., 2022b). However, direct head-to-head comparisons between aumolertinib and osimertinib are lacking. Moreover, marked ethnic differences in ILD susceptibility must be considered. Japanese populations have a substantially higher risk compared to non-Japanese populations, as evidenced by osimertinib-associated ILD rates of 8.42% versus 2.30%, respectively. Our patient is Chinese, a population with a lower baseline ILD risk than Japanese individuals. Nevertheless, he still developed grade IV ILD with osimertinib, underscoring the unpredictable nature of this complication.

Several pharmacological factors may contribute to differential pulmonary toxicity among third-generation TKIs. In preclinical studies, osimertinib exhibits approximately 200-fold greater potency against mutant versus wild-type EGFR. Aumolertinib demonstrates comparable or greater selectivity, potentially reducing off-target effects in lung tissue, although direct comparative data are limited. Both osimertinib and aumolertinib form a covalent bond with the EGFR C797 residue. However, differences in their anilino-pyrimidine scaffolds and the formation of reactive metabolites could theoretically influence tissue-specific toxicity. Osimertinib metabolism involves CYP3A4-mediated oxidation, potentially generating reactive intermediates. Whether such metabolites contribute to pulmonary injury remains speculative. A recent study suggested that osimertinib may upregulate the expression of CD40 and CD83 on dendritic cells, potentially increasing immune-related toxicity when combined with immunotherapy (Wu et al., 2023). Whether aumolertinib differentially affects dendritic cell activation is unknown but warrants investigation. Future research is needed to directly compare the incidence of ILD among third-generation TKIs in well-designed prospective studies and to elucidate the mechanistic basis for their differing pulmonary toxicity profiles.

Initiating aumolertinib at 55 mg/day aimed to reduce the initial drug exposure level, thereby minimizing potential re-stimulation of immune or toxic responses in the lungs and providing a gentler adaptation period for the body (Chen et al., 2023). This low-dose start functioned as a drug challenge test to observe whether the patient would exhibit early signs of ILD recurrence at the minimal effective dose. The absence of such signs provided preliminary evidence of drug tolerance. The standard recommended dose of aumolertinib is 110 mg once daily, established in pivotal clinical trials as the dose for optimal efficacy. Long-term use at a substandard dose carries the risk of suboptimal tumor control. After a period of 3 months of low-dose treatment, with the patient showing stable clinical symptoms, no imaging evidence of active or recurrent ILD, and manageable other adverse effects, good tolerance to aumolertinib was indicated. Subsequently, the dose was escalated to the standard 110 mg, with the goal of achieving definitive anti-tumor efficacy, while continuing nintedanib and implementing close monitoring.

Compared to previously reported cases, the present case demonstrates unique features. Among published cases of EGFR-TKI rechallenge following osimertinib-induced ILD, most involve grade I-II ILD. Reports of successful rechallenge after grade IV ILD are extremely rare (Table 1). Gu et al. reported a case of grade IV ILD where osimertinib was resumed after recovery, leading to ILD recurrence and patient death within 1 week, highlighting the extremely high risk of rechallenge in this setting (Gu et al., 2024). Yanai et al. reported a successful strategy in a patient with grade II ILD involving a switch to afatinib first, followed by a reduced dose of osimertinib (Yanai et al., 2025). Other literature documents successful cases of switching to aumolertinib after osimertinib-induced ILD (Chen et al., 2023). Our case reports the successful experience of rechallenge with aumolertinib initiated at a reduced dose (55 mg/day) and combined with adequate corticosteroids and the anti-fibrotic agent nintedanib, following grade IV ILD requiring endotracheal intubation and ICU management. The innovative aspects of this strategy include: ① Challenging the conventional view that grade IV ILD warrants permanent drug discontinuation; ② Being the first reported use of aumolertinib as the first-line rechallenge agent after grade IV ILD; ③ Employing a comprehensive management model combining dose reduction at initiation, corticosteroid consolidation, and anti-fibrotic therapy. This case provides an important reference for clinical decision-making regarding treatment options after osimertinib-induced severe ILD.

**TABLE 1:** Literature review of rechallenge with EGFR-TKIs after osimertinib-induced ILD.

Case	Age	Sex	EGFR mutation	Osimertinib dose	Time to onset	ILD grade	Rechallenge drug (dose)	Corticosteroid during rechallenge (dose)	Recurrence of ILD	References
1	38	F	L858R, T790M	80 mg	31 days	NA	Osimertinib (80 mg daily)	No	No	Mamesaya et al. (2017)
2	75	F	Exon 19 deletion, T790M	80 mg	64 days	2	Osimertinib (40 mg daily)	Yes (PSL 0.5 mg/kg daily)	No	Miyauchi et al. (2017)
3	62	M	Exon 19 deletion, T790M	80 mg	82 days	2	Osimertinib (40 mg daily)	Yes (PSL 25 mg daily)	No	Kiriu et al. (2018)
4	82	M	Exon 19 deletion, T790M	80 mg	8 months	4	Osimertinib (80 mg every other day > daily)	Yes (PSL 40 mg daily > off)	No	Nagasaka and Gadgeel (2018)
5-1	60	M	Exon 19 deletion, T790M	NA	6 weeks	3	Osimertinib (NA)	No	Yes	Nagasaka and Gadgeel (2018)
5-2	60	M	Exon 19 deletion, T790M	NA	NA	2	Osimertinib (NA)	Yes (PSL 20 mg daily > off)	No	Nagasaka and Gadgeel (2018)
6	69	F	L858R, T790M	80 mg	55 days	1	Osimertinib (40 mg daily)	Yes (PSL 10 mg daily)	No	Satoh et al. (2017)
7	57	F	Exon 19 deletion, T790M	80 mg	3 weeks	3	Osimertinib (80 mg every other day > daily)	Yes (PSL 0.5 mg/kg daily >5 mg daily)	No	Lu and Dowell (2020)
8	75	F	Exon 19 deletion, T790M	80 mg	6 months	2	Osimertinib (80 mg daily)	Yes (PSL 5 mg daily)	No	Itano et al. (2020)
9	71	F	Exon 19 deletion	80 mg	1 week	3	Osimertinib (40 mg daily>80 mg daily)	No	No	Mohammed et al. (2021)
10-1	NA	NA	NA	80 mg	155 days	1	Osimertinib (40 mg daily)	No	Yes	Nasu et al. (2020)
10-2	NA	NA	NA	40 mg	NA	1	Afatinib (30 mg daily)	No	No	Nasu et al. (2020)
11	NA	NA	NA	80 mg	43 days	2	Afatinib (40 mg daily)	No	No	Nasu et al. (2020)
12	NA	NA	NA	80 mg	51 days	3	Afatinib (20 mg daily)	No	No	Nasu et al. (2020)
13	NA	NA	NA	80 mg	23 days	1	Afatinib (40 mg daily)	No	No	Nasu et al. (2020)
14	78	F	L858R	80 mg	4.5 months	2	Afatinib (30 mg daily)	No	No	Nishima et al. (2021)
15	84	F	Exon 19 deletion	80 mg	46 days	3	Afatinib (20 mg daily)	Yes (PSL 5 mg daily > off)	No	Sato et al. (2021)
16	81	F	L858R	80 mg	44 days	3	Erlotinib (100 mg daily)	Yes (PSL 5 mg daily)	No	Fujimoto et al. (2025)
17	62	F	Exon 19 deletion	80 mg	69 days	3	Gefitinib (250 mg daily)	Yes (PSL 5 mg daily)	No	Shimbu et al. (2024)
18	82	F	Exon 19 deletion	80 mg	7 days	3	Gefitinib (250 mg daily)	Yes (PSL 15 mg daily>5 mg daily)	No	Shimbu et al. (2024)
19-1	77	M	L858R	80 mg	91 days	2	Osimertinib (40 mg daily)	No	Yes	Shimbu et al. (2024)
19-2	77	M	L858R	40 mg	7 days	2	Gefitinib (250 mg daily)	No	Yes	Shimbu et al. (2024)
20	72	F	L858R	80 mg	62 days	3	Gefitinib (250 mg daily)	No	No	Shimbu et al. (2024)
21	57	F	L858R	80 mg	15 days	2	Gefitinib (250 mg daily)	Yes (PSL 30 mg daily >2.5 mg daily)	No	Shimbu et al. (2024)
22	78	F	L858R	80 mg	51 days	3	Gefitinib (250 mg daily)	No	No	Shimbu et al. (2024)
23-1	67	F	L858R	80 mg	42 days	2	Afatinib (20 mg daily)	Yes (PSL 10 mg daily > off)	No	Yanai et al. (2025)
23-2	67	F	L858R	80 mg	42 days	2	Osimertinib (40 mg daily)	Yes (PSL 20 mg daily >15 mg daily)	No	Yanai et al. (2025)
24	56	F	Exon 19 deletion	80 mg	96 days	2	Furmonertinib (80 mg daily)	Yes (PSL 20 mg daily > off)	No	Wei et al. (2025)
25	81	F	L858R	80 mg	44 days	3	Erlotinib	No	No	Fujimoto et al. (2025)
26	64	M	L858R	80 mg	90 days	3	Aumolertinib	Yes (PSL 10 mg daily>5 mg daily > off)	No	Chen et al. (2023)
27	73	M	Exon 19 deletion	80 mg	93 days	4	Aumolertinib	No	No	Present case

This study has the inherent limitations of a single-case report. The follow-up period in this report is relatively short. The long-term success rate and safety of the rechallenge strategy require confirmation through future studies with longer follow-up and more cases. Due to ethical and practical constraints in clinical practice, we were unable to obtain ideal patient-specific biological samples. Confirming the differences between various third-generation EGFR-TKIs will require prospective collection and analysis of biological samples from more patients in the future. The primary value of this report lies in providing a detailed proof-of-concept and describing a manageable clinical pathway for a successful treatment rechallenge following osimertinib-induced grade 4 ILD, rather than in comparing drug safety or establishing a universal protocol. The systematic management approach adopted in this case offers a structured procedural framework that can be referenced and validated by other clinicians. Its reproducibility needs to be tested through the accumulation of more cases. The experience is most directly applicable to populations with similar characteristics (East Asian, EGFR sensitizing mutations). For non-East Asian populations, the management principles and the concept of cautious rechallenge remain valuable, but the specific probabilities of safety and efficacy need further confirmation in studies involving local populations. Nintedanib may have reduced the risk of an abnormal fibrotic response upon re-exposure to a TKI by inhibiting fibroblast activation and fibrotic pathways, but its precise protective mechanism and efficacy still require validation in prospective studies. The assessment of lung recovery in this case relied primarily on clinical symptoms, oxygenation indices, and imaging. The lack of pulmonary function tests limits a more precise quantification of the degree of functional recovery.

## Conclusion

In conclusion, this case demonstrates that for patients who have recovered from osimertinib-induced severe ILD following adequate pulmonary reparative therapy, a rechallenge strategy initiating with a low dose of aumolertinib followed by dose escalation may be a safe and effective treatment option. This provides a novel perspective and important practical reference for managing this clinical dilemma. Future prospective studies are needed to define the optimal timing for rechallenge, drug selection, and biomarkers predictive of success.

## Data Availability

The raw data supporting the conclusions of this article will be made available by the authors, without undue reservation.
